# Equilibration of precipitants in a counter-diffusion apparatus for protein crystallization

**DOI:** 10.1107/S1600576723004958

**Published:** 2023-06-23

**Authors:** Umberto A. Kober, Ebuka A. Ogbuoji, John A. Hutchinson, Timothy C. Mueser, Constance A. Schall

**Affiliations:** a University of Toledo, Chemical Engineering, 2801 West Bancroft Street, Toledo, OH 43606, USA; b University of Toledo, Chemistry and Biochemistry, 2801 West Bancroft Street, Toledo, OH 43606, USA; Oak Ridge National Laboratory, USA; North Carolina State University, USA

**Keywords:** microgravity, diffusion, large-volume crystals, protein crystallization

## Abstract

A cost-effective capillary dialysis apparatus (Toledo Capillary Box, TCB) developed for biomacromolecule crystal growth in microgravity and unit gravity environments can provide slow equilibration between the precipitant reservoir and capillary solutions, nurturing growth of neutron-diffraction-quality crystals. Under microgravity conditions, mass transfer of precipitants and biomacro­mol­ecules occurs under diffusion-controlled conditions, promoting slow growth and suppressing defect formation. The equilibration of common precipitants (polyethyl­ene glycol and salts such as ammonium sulfate) between capillary and reservoir solutions was measured for capillaries oriented horizontally or vertically with respect to the gravitational field at unit gravity. Precipitants equilibrated less rapidly in the vertical orientation when capillary solution densities were lower than those of the reservoir solutions. A plug filled with agarose gel was introduced in the TCB apparatus for salt precipitants since salts often exhibit relatively high free diffusion. Equilibration of the capillaries with reservoir solutions was significantly delayed for many of the salt precipitants tested. Analytical and semi-analytical models allow the prediction of precipitant equilibration of capillary and reservoir solutions under diffusion-controlled transport and show good agreement with experimental results.

## Abstract 2

A cost-effective capillary dialysis apparatus (Toledo Capillary Box) was developed for biomacromolecule crystal growth in microgravity and unit gravity environments to provide slow equilibration between the precipitant reservoir and capillary solutions. Analytical and semi-analytical models allow the prediction of precipitant equilibration of capillary and reservoir solutions under diffusion-controlled transport and show good agreement with experimental results.

## 1. Introduction

Knowledge of a biomacromolecule’s structure at the atomic level facilitates our understanding of its function. X-ray diffraction (XRD) studies can provide this, but often do not provide unambiguous placement of protons (hydrogen and deuterium). Neutron diffraction (ND) can discern deuteration/protonation states of important residues in enzyme mechanisms, ligand binding and active site residues, as well as the positions of hydrogen and deuterium atoms in protein structures (Helliwell, 2020 ▸; Blakeley *et al.*, 2008 ▸; Kono & Tamada, 2021 ▸). However, due to the relatively low flux of neutron sources, and neutron scattering from small hydrogen or deuterium atomic nuclei, large crystals (>1 mm^3^) are often required for ND data collection. The need for these large crystals makes microgravity an ideal environment for growing large, diffraction-quality crystals of target biomacromolecules (Hashizume *et al.*, 2020 ▸). Crystal defects such as solvent inclusions and anisotropic growth, apparent at unit gravity, affect the quality of ND and XRD data. The suppression of buoyancy-driven convection and sedimentation at microgravity is expected to result in improvement in crystal quality (Kundrot *et al.*, 2001 ▸; McPherson & DeLucas, 2015 ▸; Govada & Chayen, 2019 ▸).

Supersaturation, the driving force for crystal nucleation and growth, can be achieved in protein solutions by the addition of precipitants. A relatively high initial supersaturation is necessary for primary crystal nucleation in protein solutions compared with that required for small molecules (Bhamidi *et al.*, 2001 ▸, 2002 ▸; Takahashi *et al.*, 2019 ▸). In batch or semi-batch systems (*i.e.* hanging or sitting drops), this high initial supersaturation can result in rapid crystal growth of nucleated crystals, promoting incorporation of impurities, solvent inclusions or other crystal defects (Takahashi *et al.*, 2019 ▸; Schutt *et al.*, 2009 ▸; Yoshizaki *et al.*, 2006 ▸). Counter-diffusion methods have been developed to slow the approach to supersaturation, generate a spatial gradient of precipitant and grow large-volume macromolecular crystals for ND (Takahashi *et al.*, 2019 ▸; Gavira, 2016 ▸; Ng *et al.*, 2015 ▸; García-Ruiz, 2003 ▸; Tanaka *et al.*, 2004 ▸). For example, using a Granada apparatus (Triana Science and Technology), a protein solution is loaded into a capillary tube with the open end of the capillary inserted into a cross-linked gel (usually agarose). The opposing end is sealed with an impermeable plug. The precipitant solution is loaded in a reservoir in contact with the gel. Gradual diffusion of the precipitant from the reservoir solution and the protein from the capillary through the gel produces a supersaturation gradient for crystal nucleation and growth (Otálora *et al.*, 2009 ▸; García-Ruiz *et al.*, 2016 ▸; Ng *et al.*, 2015 ▸). Crystals are observed in both the gel and the capillary.

**Figure 1:**
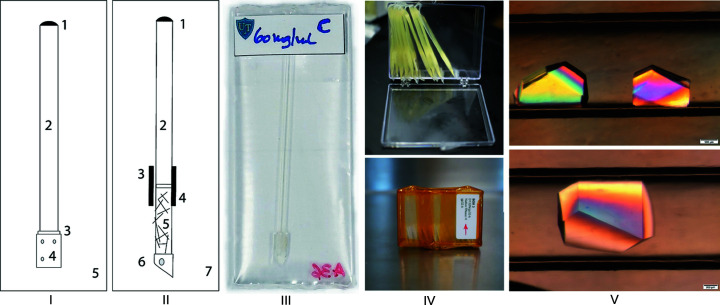
(I) Schematic of the TCB counter-diffusion apparatus, which consists of a paraffin-wax-sealed (1) capillary tube containing the crystallizing solution (2) and covered with a dialysis membrane at the opposite end (3), held in place using Tygon tubing (4). Capillaries are placed in a polythene bag (5) with reservoir (precipitant) solution, which is then sealed. (II) Schematic of the TCB counter-diffusion apparatus with the gel plug attached. The capillary tube containing the crystallizing solution (2) is sealed with paraffin wax (1). A Tygon tube (3) holds the dialysis membrane in place (4) and the capillary extension is connected (5). Another Tygon tube cut at an angle is used to allow free flow if in contact with the bag walls (6). Capillaries are placed in a polythene bag (7) with reservoir (precipitant) solution which is then sealed. (III) Photograph of a TCB-labeled bag and (IV) bags loaded in an acrylic box and packaged for flight to the ISS. (V) XI crystals grown in the TCB apparatus at unit gravity.

This work presents a study of equilibration of precipitant solutes in capillaries using a simple counter-diffusion apparatus, the Toledo Capillary Box (TCB) (Fig. 1 ▸). The TCB has been successfully used in microgravity protein crystallization experiments on the International Space Station (ISS) where large and ND-quality crystals of perdeuterated tryptophan synthase (TS) were produced (Drago *et al.*, 2022 ▸). The ensemble was assembled in an acrylic box with the dimensions 10 × 5 × 7.5 cm and a capacity of 50 sets of capillary and reservoir (precipitant) solutions. A protein solution was loaded into a capillary and retained by an ultrafiltration membrane surrounded by a precipitant reservoir solution in a polythene bag. The precipitant is transported into the capillary solution from the surrounding reservoir solution. An agarose gel plug can be affixed adjacent to the capillary membrane. The protein is retained in the capillary and does not diffuse into the gel, making the TCB unlike the Granada apparatus and other similar devices. Significant background signal can be produced by the gel matrix in ND studies for protein crystals embedded in a gel matrix. A quartz capillary tube was used for biomacromolecular crystal growth in ND studies to reduce background scattering. Hence ND data can potentially be collected without transferring crystals from the capillaries (Ng *et al.*, 2015 ▸).

Equilibration of the capillary solution (water) with polyethyl­ene glycol (PEG) or salt precipitants with the reservoir solution at unit gravity was assessed in the TCB apparatus with capillaries oriented parallel or perpendicular to Earth’s gravitational field, *i.e.* with vertical or horizontal orientation, respectively. From a list of commonly used salt precipitants in protein crystallization, six salts were chosen for study: ammonium sulfate, ammonium phosphate, lithium sulfate, sodium chloride, sodium potassium tartrate and sodium malonate (McPherson, 2004 ▸). Analytical and semi-analytical models were employed to estimate effective diffusion coefficients of precipitants in water and agarose gels for the diffusion-controlled conditions of the capillaries, valid only for a vertical capillary orientation. The effect of reduced precipitant equilibration rate on d-xylose isomerase (XI) crystallization using an agarose plug was explored. The models can be used to develop strategies to achieve slow precipitant equilibration in a counter-diffusion apparatus and to inform decisions for sample loading for launch to the ISS to ensure initiation of crystal growth primarily under microgravity conditions.

## 2. Materials and methods

### 2.1. Capillary dialysis apparatus

For these studies, glass borosilicate capillary tubes were used (Kimble Chase 34500–99) with inner and outer diameters of 1.5 and 1.8 mm, respectively, cut to a length of 4.9 cm. One end of the capillary tube was covered with a cellulose acetate dialysis membrane, with a molecular weight cut-off (MWCO) of 8–10 kDa (Spectrum Labs, New Brunswick, NJ, USA) for salt and PEG precipitants. The PEG 8000 transport across the 8–10 kDa membrane was found to be similar to that of higher MWCO membranes (see Section S1 of the supporting information). The membrane was held in place using a perforated flexible Tygon tube with an internal diameter of 2 mm (Fischer Scientific, Pittsburg, PA, USA). The Tygon tube is perforated along its perimeter to ensure contact of the reservoir solution with the membrane after placement in the polythene bag. The empty assembly was then placed in a water (or buffer) filled beaker to assess potential membrane damage and leakage of solution into the capillary. Subsequently, capillary solution was injected into the capillary tube using a blunt-end syringe needle at the open end. The open end of the capillary was sealed using a minimal amount of paraffin wax, which rapidly solidifies on contact with the capillary walls without impacting the internal temperature, and was placed in a polythene bag with reservoir (precipitant) solution before sealing with a heat sealer. This process was successfully used for production of protein crystals for ND under microgravity conditions as described by Drago *et al.* (2022 ▸). The reservoir and capillary volumes were 2 ml and 87 µl, respectively. The capillary tubes were oriented vertically or horizontally with respect to the Earth’s gravitational field. In the vertical position, the sealed end of the capillary tube was oriented towards the top end of the bag.

For studies using an agarose gel plug, a second capillary tube (1–2 cm in length) was positioned below the dialysis membrane, filled with agarose gel (USB Corporation, Cleveland, OH, USA) and held in place using a Tygon tube. A solution of 3%(*w*/*v*) agarose in deionized water was heated to 90°C and stirred for 20 min at 300 rev min^−1^. The gel was loaded into the glass capillary with a blunt needle syringe and cooled to room temperature. With the addition of the plugs, a longer bag with a reservoir volume of 3 ml was used.

### 2.2. Capillary and reservoir solutions

Deionized water was used in all capillary solutions for the equilibration experiments. The polymeric reservoir solutes included PEG 4000 and 8000 (Hampton Research, Aliso Viejo, CA, USA), and PEG 400 (Fluka Chemical, Milwaukee, WI, USA). The PEG reservoir solution concentrations were kept at 15%(*w*/*v*) except for PEG 400 which was 15%(*v*/*v*). All salt reservoir solutions were kept at 10%(*w*/*v*) concentration. The salt precipitants include sodium malonate and lithium sulfate (MP Biomedicals, Irvin, CA, USA), sodium chloride (Sigma–Aldrich, St Louis, MO, USA), sodium potassium tartrate, ammonium phosphate and ammonium sulfate (Fischer Scientific, Pittsburg, PA, USA).

### 2.3. Equilibration studies

Precipitants in the reservoir solutions were allowed to equilibrate with the capillary solutions between 5 and 720 h. All setups were placed in an incubator at a temperature of 20°C, unless otherwise noted. The specific gravity of the precipitant solutions was greater than that of the capillary solution (water) for all experimental conditions.

A volume average capillary concentration was measured by extracting the entire capillary solution sample, then mixing the sample and measuring the refractive index (RI) in a Milton Roy Abbe refractometer at room temperature (22 ± 2°C). Standard curves were developed for all precipitants, and the RIs for reservoir solutions were found to vary linearly over the precipitant concentration range. The volume average capillary concentration was measured in triplicate for each equilibration time.

### 2.4. Protein crystallization

Solutions of *Streptomyces rubiginosus* XI (Genencor, Rochester, NY, USA) were prepared through buffer exchanges using 10 kDa MWCO Amicon membrane concentrators. Three exchanges of the protein solution were made with 0.1 *M* tris­(hy­droxy­methyl)­amino­methane pH 8.0 (Tris–HCl buffer), containing 10 m*M* ethyl­enedi­amine­tetra­acetic acid (EDTA) to remove metal ions. EDTA was then removed by three exchanges with 0.1 *M* Tris–HCl buffer containing 10 m*M* MgCl_2_. XI was crystallized in the TCB apparatus at 4°C with an initial protein concentration of 40 or 60 mg ml^−1^ loaded into the 4.9 cm-long capillary tubes. The XI concentration was measured by UV absorbance at 280 nm using an extinction coefficient of 1 ml mg^−1^ cm^−1^ (Waltman *et al.*, 2014 ▸). The reservoir solutions consisted of 10%(*w*/*v*) ammonium sulfate in Tris–HCl buffer with MgCl_2_. Capillaries were orientated vertically with respect to the gravity field at unit gravity. Five replicates of each experimental condition [protein concentration, no agarose plug, 1 and 2 cm plug length filled with 3%(*w*/*v*) agarose] were incubated for 28 days at 4°C (Table 1 ▸). Using an optical microscope, the lengths of the two longest visible dimensions of the three largest crystals were measured and subsequent areas were calculated.

## 3. Results and discussion

### 3.1. Capillary orientation and precipitant equlibration rate

**Figure 2:**
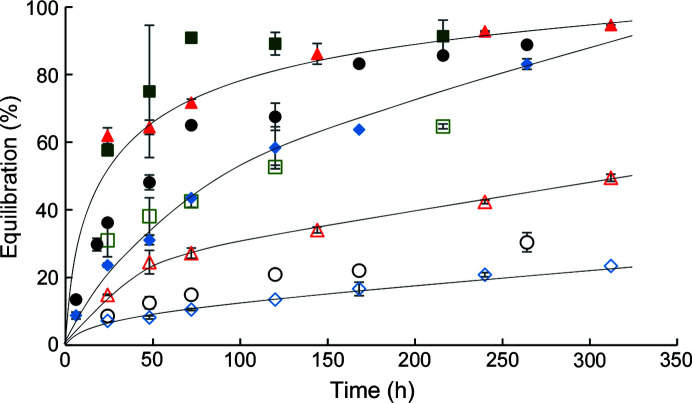
Capillary equilibration at 20°C as a function of time for ammonium sulfate (squares), PEG 400 (triangles), PEG 4000 (circles) and PEG 8000 (diamonds) for a capillary length of 4.9 cm. Filled and open symbols indicate capillaries in the horizontal and vertical orientations, respectively. Lines are provided to show the trends in data.

Equilibration of the capillary solution (water) with the surrounding ammonium sulfate or PEG reservoir solution was monitored as a function of time, with the capillaries oriented either parallel (vertical) or perpendicular (horizontal) to the Earth’s gravitational field. Percentage equilibration was expressed as the ratio of the volume average concentration of solute (precipitant) in the capillary to the initial precipitant concentration in the reservoir solution. Equilibration was found to be much more rapid in the horizontal than the vertical orientation (Fig. 2 ▸).

Buoyancy-driven convection appears to contribute to solute transport in capillaries oriented horizontally, hastening equilibration, as observed for PEG and ammonium sulfate precipitants (Fig. 2 ▸). As previously reported, a precipitant solution of higher specific gravity than the capillary solution can create density gradients and buoyancy-driven convection when the capillary is positioned such that the unsealed end is exposed to the precipitation solution at a higher point with respect to the gravitational field (Moreno & Soriano-García, 1999 ▸). In the TCB apparatus, the density gradients can form across the relatively wide diameter (1.5 mm) of capillaries oriented in the horizontal direction.

Solute transport in capillaries oriented in the vertical direction is expected to be controlled by a slower diffusive process. When capillary and reservoir solution densities match, solute equilibration is expected to be a diffusion-controlled process for all orientations, resulting in similar rates of equilibration for vertical and horizontal orientations.

For the equilibration in capillaries oriented in the vertical direction, an analytical model (Carslaw & Jaeger, 1947 ▸) describing one-dimensional diffusion of solute is given in dimensionless form as

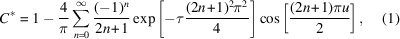

with the dimensionless concentration expressed as 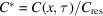 . *C*(*x*, τ) is the concentration of the solute (precipitant) in the capillary solution as a function of axial position *x*, expressed in dimensionless form *u* = *x*/*L*, where *L* is the length of the capillary tube. *C*_res_ is the concentration of solute in the reservoir solution. Equilibration time *t* is expressed in dimensionless form as τ = *Dt*/*L* ^2^, where *D* is the diffusion coefficient of the solute. The initial condition at τ = 0 is C* = 0 for 0 ≤ *u* ≤ 1.0. Assuming the reservoir concentration remains constant at the dialysis membrane interface, the boundary condition at *u* = 0 is C* = 1.0. At the sealed end of the capillary (*x* = *L*), no flux of solute occurs, and the boundary condition is 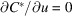 .

**Figure 3:**
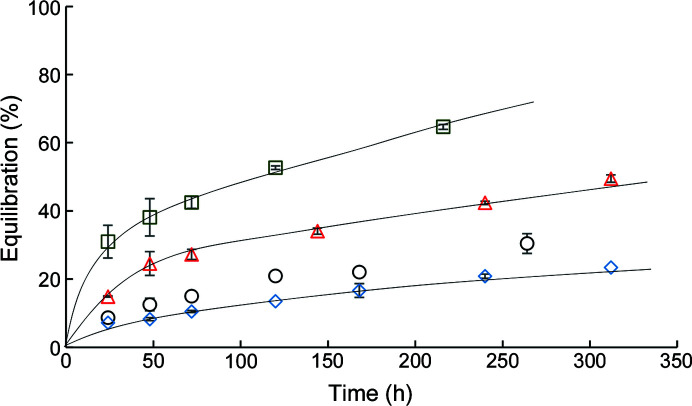
Experimental percentage equilibration as a function of time for PEG 400 (triangles), PEG4000 (circles), PEG 8000 (diamonds) and ammonium sulfate (squares) with vertical capillary orientation parallel to the gravitational field. Lines represent the percentage equilibration predicted using equation (1).

The diffusion coefficient *D* is treated as an adjustable parameter. With the value *D*, the concentration of solute in the capillary as a function of axial position and time can be modeled using equation (1). From this, a volume average concentration 〈*C*_mod_〉 can be calculated and compared with the experimentally measured volume average capillary concentration at varying equilibration times. The diffusion coefficient *D*_mod_ was obtained from the analytical model for reservoir solutions by minimizing the sum of squared errors between 〈*C*_mod_〉 and the experimentally measured volume average capillary concentration. Diffusivity increases as molecular size decreases, resulting in shorter equilibration times for PEG 400 and ammonium sulfate compared with PEG 4000 and 8000 (Fig. 3 ▸). The analytical model appears to represent the experimentally measured percentage equilibration well for all solutions, which suggests that precipitant equilibration in the vertical orientation is a purely diffusive process. Similar one-dimensional diffusion models have been used to describe temporal concentration profiles in capillary devices using the gel acupuncture or gel-tube method (Tanaka *et al.*, 2004 ▸; García-Ruiz *et al.*, 1999 ▸; Otálora & García-Ruiz, 1996 ▸; Carotenuto *et al.*, 2002 ▸).

To further assess the validity of the analytical equilibration model, the experimentally derived diffusion coefficient for samples in the vertical orientation, *D*_mod_, was compared with previously reported infinite dilution diffusion coefficients or those derived from empirical correlations when published values could not be found (Table 2 ▸) for PEG and salt precipitants.

For PEG precipitants, diffusion coefficients at infinite dilution and 25°C were estimated from a correlation [equation (2)] developed for high-purity monodisperse PEG with molecular weights between 300 and 4000 Da (Johansson *et al.*, 1991 ▸), where 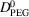 is the diffusion coefficient (m^2^ s^−1^) and *M*_w_ is the molecular weight of PEG (g mol^−1^):

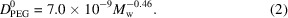

The following equation can be used to estimate the overall diffusion coefficient, *D*_0_, of salt solutes in water (Cussler, 2009 ▸):

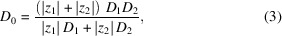

where *z*_1_ and *z*_2_ are the ionic charges and *D*_1_ and *D*_2_ are the ionic diffusion coefficients for the cation and anion, respectively.

The diffusion coefficients were corrected for the equilibration experimental temperature using (Cussler, 2009 ▸)

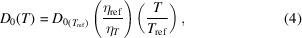

where *T* is the absolute temperature and the data for water viscosity η, at reference and experimental temperatures, were used for all aqueous precipitant (reservoir) solutions.

For ammonium sulfate, *D*_mod_ is approximately equal to a previously published value (Mohan *et al.*, 2000 ▸). Lithium sulfate and sodium chloride had *D*_mod_ values which differ from the published values by 5.7 and 14.1%, respectively (Wang *et al.*, 2016 ▸; Araki & Arai, 1967 ▸). For sodium malonate and ammonium phosphate, *D*_mod_ differed from the calculated values by 4.3 and 10.5%. Published values for sodium potassium tartrate or its anion were not found.

Diffusion coefficients obtained from the analytical model and estimated from empirical correlations differed by less than 3% for PEG 400 but deviated by ∼20% for PEG 4000 and ∼38% for PEG 8000 (Table 2 ▸). The range of PEG molecular weight used in the derivation of the empirical correlation [equation (2)] was between 300 and 4000 Da for monodisperse PEG (Johansson *et al.*, 1991 ▸). The molecular weight of PEG 400 falls within this range, whereas PEG 4000 is at the limit of the range and PEG 8000 is outside the range. The deviations between modeled and calculated diffusion coefficients for PEG 8000 may be due to the lack of validity of the empirical correlation [equation (2)] for higher *M*_w_ PEG. Deviations could also arise, in part, from the polydispersity or impurity of PEG used in equilibration experiments (Johansson *et al.*, 1991 ▸). Higher measured diffusion rates can occur in polydisperse PEG solute, particularly at initial equilibration times. Buffer salts added to the PEG precipitant solution for crystallization experiments may increase solution viscosity and hence diffusion. However, for salts this viscosity increase only appears to be significant at high molarity (0.5 to 1 *M*) with solution viscosity dominated by PEG concentration for ternary mixtures (Mei *et al.*, 1995 ▸).

The results show overall satisfactory agreement between the modeled parameter *D*_mod_ and reported values of free diffusion from the literature or estimated values from empirical equations. The results support the hypothesis of a purely diffusive process for the capillary precipitant equilibration with the reservoir solution for capillary tubes placed in a vertical orientation (parallel to the gravitational field).

### 3.2. Semi-analytical model for multilayer diffusion of salt precipitants through an agarose plug

Salts often exhibit higher free diffusion than polymer precipitants due to their smaller particle sizes [*i.e.* ionic radius for salts in solution (Kienle & Schwartz, 2019 ▸)]. Insertion of a hydro­gel (*i.e.* agarose) plug between the capillary membrane and the reservoir solution offers a means to delay equilibration of the capillary solution with the reservoir solution by decreasing precipitant effective diffusivity in the gel plug. The gel network can interact with the solute cation or anion and obstruct the diffusing solute.

**Figure 4:**
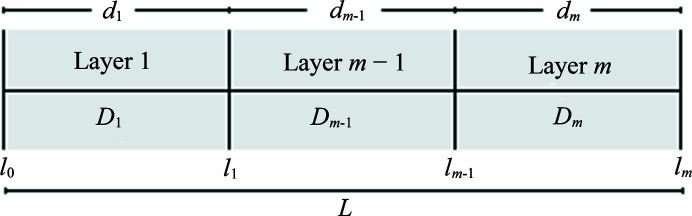
One-dimensional composite slab with axial position *x*. The interval *l*_0_ < *x* < *l*_*m*_ consists of *m* layers. The interfaces between adjacent layers are located at *x* = *l_i_* (*i* = 1, …, *m* − 1), the width of layer *i* is denoted as *d_i_* = *l_i_* − *l*_*i*−1_ and the diffusivity in layer *i* is denoted as *D_i_*. External boundary conditions apply at the ends of the slab (at *x* = *l*_0_ and *x* = *l_m_*) with internal boundary conditions defined at the interfaces (Carr & Turner, 2016 ▸).

The effective diffusion coefficient of the solute in the agarose gel can be assessed by measurement and modeling capillary equilibration of salt precipitants with an inserted agarose plug as a function of time. With the addition of the gel plug to the TCB apparatus, the transport model must be modified to account for multi-layer one-dimensional diffusion using a semi-analytical solution method (Carr & Turner, 2016 ▸). Each layer was assumed to be homogeneous and isotropic with a constant diffusivity (Fig. 4 ▸).

The governing equation for one-dimensional diffusion, defined for each layer *l*_*i* − 1_ < *x* < *l*_*i*_, is

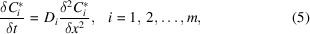

where 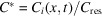 and *C*_*i*_(*x*, *t*) is the concentration of the solute (precipitant) in the capillary solution as a function of capillary axial position *x* and equilibration time *t*.

The initial condition at *t* = 0 for 0 ≤ *x* < *L* is *C*_*i*_(*x*, *t*) = 0. The boundary conditions at each end of the capillary are the same as those for the analytical model [equation (1)], with constant precipitant concentration assumed at the gel plug interface and no flux at the sealed end of the capillary. The internal interfaces are treated as perfect contacts, with boundary conditions of

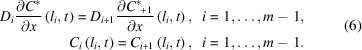

Initially, the solute diffusion coefficient in water was determined using experimentally measured equilibration data without an agarose plug and analytical model [equation (1)]. The diffusion coefficient *D*_g_ for solutes in the agarose gel layer was then obtained by solving the semi-analytical model numerically [equation (5)] (Carr & Turner, 2016 ▸) and minimizing the sum of squared errors between the experimental and modeled volume average capillary concentration.

### 3.3. Effective diffusivity of salt precipitants in agarose gel

The effective diffusion coefficient *D*_g_ decreased for most salt precipitants in the 3%(*w*/*v*) agarose gel compared with free diffusion *D*_0_. The ratio of this reduction, *D*_g_/*D*_0_, varies from 0.65 to 0.94 (Table 3 ▸). Ammonium sulfate and ammonium phosphate showed the highest reduction for *D*_g_. Sodium chloride exhibited a *D*_g_ close to *D*_0_.

Solutes with large cation radii appear to have greater decreases in effective diffusivity through the agarose gel, with the exception of sodium potassium tartrate. Increasing cation radii can impose steric hindrance for solute diffusion.

Agarose is a heterogeneous gel where the polymer chains are considered immobile at the molecular level (Amsden, 1998 ▸). To gain insights into the mechanism or factors that impact solute diffusion through the agarose matrix, Phillips, Mackie and Ogston models of solute diffusion through polymer gels were examined. The Phillips model considers hydro­dynamic effects on solute diffusion where the polymer chain increases the frictional drag on the solute diffusion near the gel polymer (Amsden, 1998 ▸); it is expressed as (Phillips *et al.*, 1989 ▸)

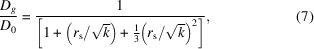

where *D*_g_ is the diffusion in the gel, *D*_0_ is the diffusion coefficient of the solute at infinite dilution, *r*_s_ is the radius of the solute given by 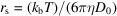 , *k* is the hydro­dynamic permeability of the medium given by 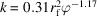 , *r*_f_ is the polymer radius and φ is the volume fraction of polymer gel.

In the Mackie and Ogston models, obstruction effects are considered where the hydro­gel acts as a stationary barrier that obstructs the solute diffusion. The Mackie model is expressed as (Mackie & Meares, 1955 ▸)

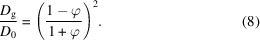

The Ogston model is expressed as (Ogston *et al.*, 1973 ▸)

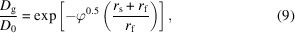

where *r*_f_ is the radius of the polymer.

The predicted effective diffusivity *D*_g_, using the Phillips model, is nearly the same as the free diffusivity for all solutes (Table 4 ▸). It appears that the hydro­dynamic mechanism does not influence the diffusion process in the agarose gel, which is probably due to the low volume fraction of polymer in the gel.

The obstruction models predict a decrease in *D*_g_. Mackie’s model only considers the volume fraction of the polymer and predicts values higher than those experimentally measured with the exception of sodium chloride and lithium sulfate (Mackie & Meares, 1955 ▸). The Ogston model shows better agreement with the experimental values of *D*_g_ than the Mackie model for most solutes. The calculated values are close to the experimental values for many solutes but higher for ammonium sulfate and ammonium phosphate, and lower for sodium chloride and lithium sulfate. For the Ogston model, the gel polymer chain and solute radii are also considered (Ogston *et al.*, 1973 ▸). Obstruction appears to be a significant factor in salt diffusion in the agarose gel.

Electrostatic effects may also contribute to the lower experimental *D*_g_ observed. Although agarose is often considered to be a neutral polymer, charged impurities along its backbone such as sulfonates, ester sulfates, ketals, carboxyls and predominately pyruvates have been reported (Dumitriu, 2004 ▸; Wang *et al.*, 2016 ▸). Small quantities of sulfonate and pyruvate can give the gel a slight but significant negative charge (Lead *et al.*, 2003 ▸; Johnson *et al.*, 1995 ▸). These impurities can affect the physicochemical properties of the gel and intermolecular interactions between charged solutes and the agarose polymer (Araki & Arai, 1967 ▸). The negatively charged groups in the gel can repel anions and enrich the gel with cations due to electrostatic interactions (Fatin-Rouge *et al.*, 2003 ▸). Charged groups present in agarose gels have previously been reported to decrease the mobility of cations and anions in the gels, suggesting that local intermolecular interactions can contribute to the observed decreased diffusivity of ionic species in the gel (Fatin-Rouge *et al.*, 2004 ▸; Golmohamadi *et al.*, 2012 ▸). The strength of the ionic interactions is expected to vary depending on the electronegativity and electropositivity of the ionic species.

The intermolecular interactions between the gel and the ionic solute are neglected in the models examined in Table 4 ▸. Although the diffusion mechanisms of salts in heterogeneous gels such as agarose are not entirely clear, the models and results indicate that obstruction and ionic radii impact *D*_g_. Ionic interactions that are not captured in the models examined may also influence *D*_g_.

### 3.4. Protein crystallization and precipitant equlibration rate

**Figure 5:**
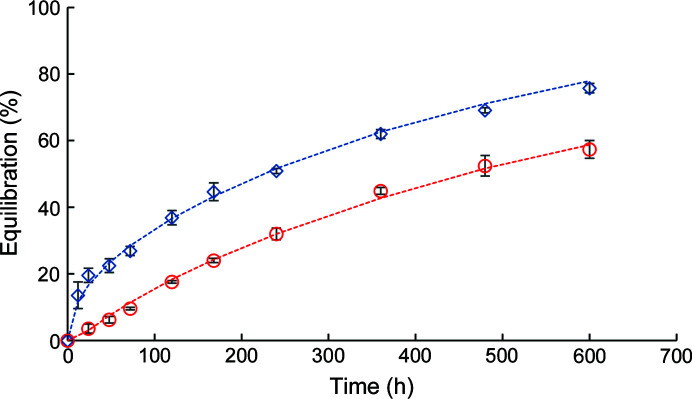
Equilibration at 4°C of 10%(*w*/*v*) reservoir solutions of ammonium sulfate with capillary tubes of 4.9 cm length oriented in the vertical direction as a function of equilibration time. An 8–10 kDa MWCO membrane separates the capillary and reservoir solutions. Symbols are averages from experimental measurements and the error bars represent one standard deviation for no gel plug (diamonds) and a 1 cm 3% agarose gel plug (circles). Lines represent the percentage equilibration modeled for diffusive transport of precipitant from the reservoir to the capillary.

**Figure 6:**
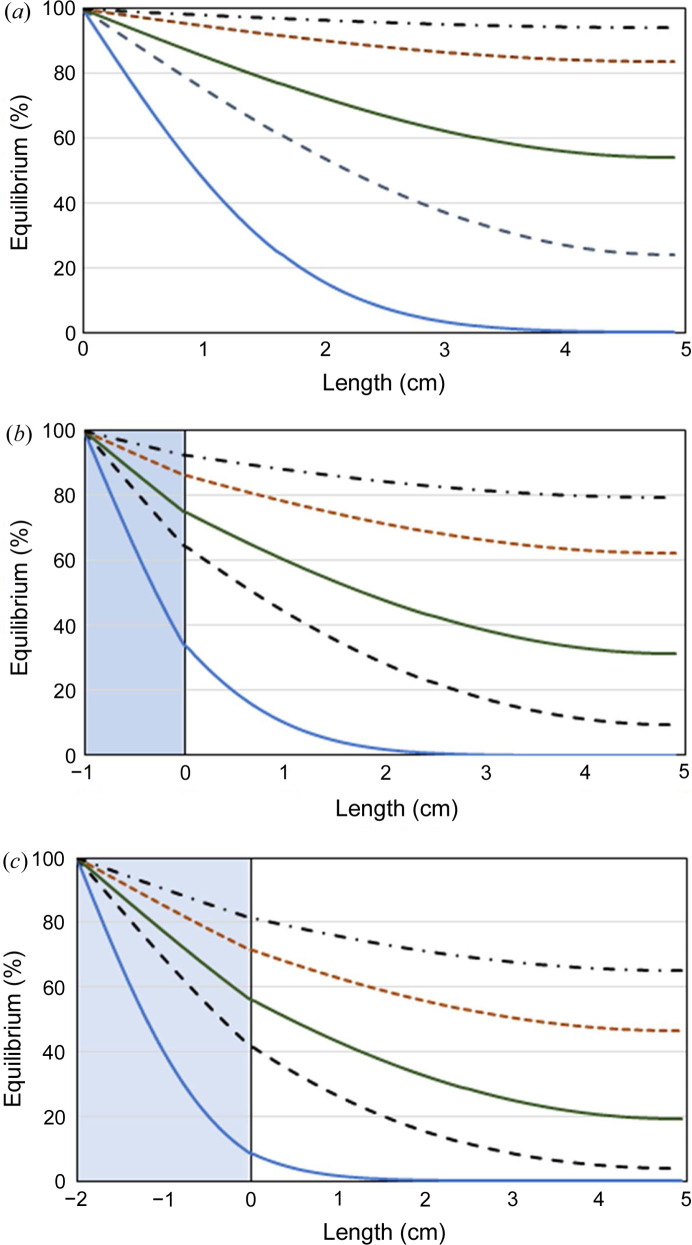
Simulated temporal capillary precipitant equilibration for one-dimensional diffusion as a function of axial position (*L*) for an ammonium sulfate reservoir solution at 4°C. The capillary length is 4.9 cm between the membrane and sealed end. The negative length indicates the position in the gel plug. (*a*) No gel plug; (*b*) 1 cm 3% agarose gel plug; (*c*) 2 cm 3% agarose gel plug. Equilibration times: 24 h (blue ––); 120 h (– – –); 240 h (green ––); 480 h (- - -); 720 h (– · –).

Agarose gels can be used to decrease the diffusivity of some salt precipitants (Table 4 ▸) and produce a slower approach to precipitant equilibration between the capillary and reservoir solutions in a counter-diffusion apparatus (Fig. 5 ▸). Equilibration profiles can be simulated using *D*_0_ and *D*_g_ to predict precipitant equilibration as illustrated for ammonium sulfate at 4°C with *D*_0_ = 5.76 × 10^−6^ m^2^ s^−1^ and *D*_g_ = 4.98 × 10^−6^ m^2^ s^−1^ (Fig. 6 ▸).

The effect of precipitant equilibration on protein crystal size was evaluated for the crystallization of XI at 4°C with an ammonium sulfate precipitant in the buffer. Capillaries with lengths of 4.9 cm were prepared. Two lengths of 3%(*w*/*v*) agarose gel plugs (1 and 2 cm) were added to sets of capillary tubes. Protein solutions with initial concentrations of 40 or 60 mg ml^−1^ were loaded into capillaries with and without gel plugs with five replicates for each condition (Tables 1 ▸ and 5 ▸).

Qualitatively, there appeared to be less aggregation of crystals in capillary tubes with gel plugs. In tubes without a gel plug, a mass of aggregated crystals was observed near the membrane. In all conditions, most crystals appear to form near the membrane and not further than 2 cm from the membrane. This suggests a limited crystal nucleation zone inside the capillary chamber.

A fast equilibration of the capillary solution with the reservoir ammonium sulfate precipitant solution is observed in simulated and experimental data for capillary tubes without a gel plug (Figs. 5 ▸ and 6 ▸). A high concentration of precipitant near the membrane is predicted to rapidly form inside the capillary chamber. This higher concentration can lead to excessive nucleation followed by rapid crystal growth near the membrane as exhibited by the aggregation of crystals near the membrane.

The crystal size was reported as the crystal area through measurement of the two largest dimensions of the three largest crystals in each capillary tube (Table 5 ▸). A two-factor analysis of variance (ANOVA) was conducted to assess the significance of capillary conditions, initial protein concentration and gel plug length, on the crystal size. Protein concentration and plug length significantly affected crystal size with *P* values of 2.16 × 10^−4^ and 7.82 × 10^−6^, respectively. The interaction between these factors was not significant (*P* = 0.42).

The ANOVA was followed by a Fisher least significant difference (LSD) pairwise comparison to assess the combined effect of protein concentration and the gel plug on the crystal size (expressed as surface area). The analysis showed three distinct groups (Table 6 ▸). The largest crystal sizes were found in capillaries with the highest initial protein concentration (60 mg ml^−1^) when gel plugs were inserted into the capillary tubes (group A). This was followed by the second group (B) with the initial protein concentration 40 mg ml^−1^, also with the gel plugs. Crystals grown in capillaries with the protein concentration 40 mg ml^−1^ and a 1 cm plug are also found in the group A, yielding the largest crystal sizes. Crystals of smaller sizes were found primarily where no plug gel was used (group C). The use of the gel plug appears to yield significantly larger crystal sizes.

This result, combined with analytical and equilibration data, suggests that slower precipitant equilibration of the capillary and reservoir solutions can produce larger protein crystals with well defined edges, smooth surfaces and fewer crystal clusters (see Section S4 of the supporting information), which aligns with previously reported observations (Carotenuto *et al.*, 2002 ▸). The addition of agarose plugs to the TCB appears to be a feasible method to delay crystal nucleation on the launchpad for microgravity experiments and to produce larger crystals under unit and microgravity conditions for those precipitants with decreased diffusivity in gels.

## 4. Conclusions

Equilibration of precipitant reservoir solutions in the TCB apparatus was observed to be significantly slower in capillaries oriented vertically with respect to the gravitational field compared with capillaries oriented horizontally. Diffusion appears to be the dominant transport phenomenon in the vertically oriented capillaries while density-driven convection appears to occur in the horizontal orientation. Reservoir and capillary solutions with equal densities are expected to equilibrate at rates independent of orientation with respect to a gravitational field. An analytical model for the diffusive equilibration process for the vertical orientation was developed. The model can be used to predict temporal changes in precipitant concentration profiles in the TCB apparatus prior to launch.

The use of a plug filled with agarose gel inserted between the capillary and reservoir solutions can slow precipitant equilibration between reservoir and capillary solutions. This slow precipitant equilibration can produce conditions that are more favorable for growth of the larger protein crystals required for ND structural studies.

## Supplementary Material

Supporting figures and table. DOI: 10.1107/S1600576723004958/ei5096sup1.pdf

## Figures and Tables

**Table 1:** XI crystallization conditions: initial protein concentration in the capillary and agarose gel plug 3%(*w*/*v*) in the TCB apparatus

Condition 40-0	XI concentration 40 mg ml^−1^ and no gel plug
Condition 40-1	XI concentration 40 mg ml^−1^ and 1 cm agarose plug
Condition 40-2	XI concentration 40 mg ml^−1^ and 2 cm agarose plug
Condition 60-0	XI concentration 60 mg ml^−1^ and no gel plug
Condition 60-1	XI concentration 60 mg ml^−1^ and 1 cm agarose plug
Condition 60-2	XI concentration 60 mg ml^−1^ and 1 cm agarose plug

**Table 2:** Experimentally determined diffusion coefficients of solutes (*D*_mod_) in water compared with the literature or calculated values (*D*_0_) at 20°C

Solute	*D*_mod_ × 10^10^ (m^2^ s^−1^)	*D*_0_ × 10^10^ (m^2^ s^−1^)
Ammonium sulfate (NH_4_)_2_SO_4_	11.4	11.47 (Mohan *et al.*, 2000 ▸)
Ammonium phosphate (NH_4_)_3_PO_4_	9.23	8.26†
Sodium potassium tartrate KNaC_4_H_4_O_6_·4H_2_O	9.90	
Sodium malonate C_3_H_2_O_4_Na_2_	10.2	9.76†
Sodium chloride NaCl	16.4	14.08 (Robinson & Stokes, 1955 ▸)
Lithium sulfate Li_2_SO_4_	8.27	7.8 (Leaist & Goldik, 2001 ▸)
PEG 400	4.00	3.89‡
PEG 4000	1.65	1.35‡
PEG 8000	1.35	0.98‡

† Calculated using equation (3). Values for *D*_1_ and *D*_2_ are given in Section S2 of the supporting information.

‡ Calculated using equation (2).

**Table 3:** Free diffusion of solutes in aqueous solutions (*D*_0_) and effective diffusion coefficients in 3%(*w*/*v*) agarose (*D*_g_) at 20°C

	Cation			Anion					
Precipitant	Species	*M*_w_	Radius (pm)†	Species	*M*_w_	Radius (pm)†	*D*_0_ (10^−10^ m^2^ s^−1^)	*D*_g_ (10^−10^ m^2^ s^−1^)	*D*_g_/*D*_0_
Ammonium sulfate	NH_4_^+^	18.04	151	SO_4_^2−^	96.06	244	11.44	7.52	0.65
Ammonium phosphate	NH_4_^+^	18.04	151	PO_4_^3−^	94.97	238	9.23	6.24	0.67
Sodium malonate	Na^+^	22.98	116	C_3_H_2_O_4_^2−^	102.07	258	10.20	8.55	0.84
Sodium potassium tartrate	Na^+^, K^+^	22.98, 39.09	116, 152	C_4_H_4_O_6_^2−^	148.07	275	9.90	8.39	0.85
Lithium sulfate	Li^+^	6.94	73	SO_4_^2−^	96.06	244	8.27	7.36	0.89
Sodium chloride	Na^+^	22.98	116	Cl^−^	35.45	167	16.40	15.40	0.94

† Shannon (1976 ▸); Jenkins & Thakur (1979 ▸).

**Table 4:** Experimental diffusion coefficients in aqueous solutions (*D*_0_) and in 3%(*w*/*v*) agarose gel (*D*_g_) at 20°C, and *D*_g_ estimated from diffusion models

	Experimental		Calculated *D*_g_ (10^−10^ m^2^ s^−1^)		
Precipitant	*D*_0_ (10^−10^ m^2^ s^−1^)	*D*_g_ (10^−10^ m^2^ s^−1^)	Phillips model	Mackie model	Ogston model
Ammonium sulfate	11.40	7.52	11.44	10.20	9.64
Ammonium phosphate	9.23	6.58	9.23	8.21	7.78
Sodium malonate	10.20	8.55	10.20	9.07	8.60
Sodium potassium tartrate	9.90	8.39	9.90	8.81	8.34
Lithium sulfate	8.27	7.36	8.27	7.35	6.97
Sodium chloride	16.40	15.40	16.40	14.6	13.80

**Table 5:** Average area of the three largest XI crystals and one standard deviation for each experimental condition The data for each capillary can be found in S3 of the supporting information.

Condition [protein conc. (mg ml^−1^), gel plug (cm)]	Average area (mm^2^)		Condition [protein conc. (mg ml^−1^), gel plug (cm)]	Average area (mm^2^)
40, 0	0.61 ± 0.36		60, 0	1.09 ± 0.49
40, 1	1.17 ± 0.53		60, 1	1.68 ± 0.74
40, 2	1.41 ± 0.60		60, 2	1.83 ± 0.63

**Table 6:** Fisher LSD pairwise comparison of the effect of initial protein concentration and insertion of an agarose plug between the reservoir and capillary solution on protein crystal size expressed as the mean value (mm^2^)

Condition	*N*	Mean	Grouping		
60-2	15	1.83	A		
60-1	15	1.68	A		
40-1	15	1.41	A	B	
40-2	15	1.17		B	
60-0	15	1.09		B	C
40-0	15	0.61			C
