# Supplementary material for: Equilibration of precipitants in a counter-diffusion apparatus for protein crystallization
Source: J Appl Crystallogr. 2023 Jun 23;56(Pt 4):1057–65. doi: 10.1107/S1600576723004958 (PMC10405592; doi:10.1107/S1600576723004958)
Supplement: Supplementary file 1 [file j-56-01057-sup1.pdf]

Appl Cryst JAC JOURNAL OF APPLIED CRYSTALLOGRAPHY

Volume 56 (2023)

Supporting information for article:

Equilibration of precipitants in a counter diffusion apparatus for protein crystallization

Umberto A. Kober, Ebuka A. Ogbuoji, John A. Hutchinson, Timothy C. Mueser and Constance A. Schall

S1. Effect of membrane molecular weight cut off (MWCO) on equilibration rate of PEG 8000

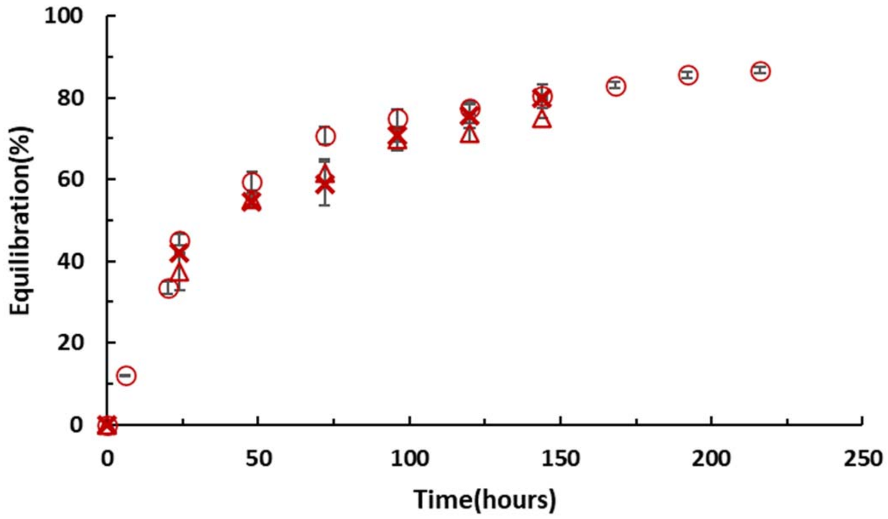

Figure S1.1. Equilibration as a function of time using membrane MWCO of 8 to 10kDa (**Δ**), 12kDa (**o**), 20kDa (×) for PEG 8000 at room temperature in the horizontal orientation.

S2. Diffusion coefficients of hydrated inorganic cations and anions

Table S2.1 Values of diffusion coefficients of hydrated inorganic cations and anions in water, at T=298.15 °K and infinite dilution. †

Hydrated metal ion	*D* (10^{-10} $m^2.s^{-1}$)	Hydrated anion	*D* (10^{-10} $m^2.s^{-1}$)
K^+	19.57	OH^-	52.7
Li^+	10.29	Cl^-	20.3
Na^+	13.34	PO_4^{3-}	6.1
NH_4^+	19.57	SO_4^{2-}	10.7

Table S2.2 Values of diffusion coefficients of Carboxylic acids in water, at T=298.15 °K and infinite dilution. †

Carboxylic acids	*D* (10^{-10} $m^2.s^{-1}$)
Malonate^{2-}	8.45[a,b]
Tartrate^{2-}	7.94[a,b]

† Henry V. K. (Ed.) *CRC Handbook of Thermophysical and Thermochemical Data*. CRC press Inc. Boca Raton, **1994**.

S3. Measured crystal areas.

Table S3.1 Areas of three largest XI crystals observed in each capillary after 28 days incubation at 4 °C using conditions outlined in Table 1. Average area and one standard deviation in each capillary are also tabulated.

Condition	Crystal Area (mm^2)			Average Area (mm^2)
40-0	0.61	0.4	0.24	0.42 ± 0.19
	0.44	0.26	0.44	0.38 ± 0.10
	0.76	0.74	0.45	0.65 ± 0.17
	1.22	0.27	0.18	0.56 ± 0.58
	1.27	1.12	0.79	1.06 ± 0.25
40-1	1.66	1.57	1.26	1.50 ± 0.21
	1.73	0.91	0.67	1.10 ± 0.56
	2.03	1.55	1.24	1.61 ± 0.40
	1.36	1.00	0.89	1.08 ± 0.25
	2.84	1.29	1.24	1.79 ± 0.91
40-2	2.07	0.67	0.52	1.09 ± 0.85
	0.84	0.57	0.31	0.57 ± 0.27
	1.19	0.99	0.98	1.05 ± 0.12
	2.23	1.74	1.10	1.69 ± 0.57
	2.05	1.45	0.82	1.44 ± 0.62
60-0	0.83	0.49	0.33	0.55 ± 0.25
	1.96	0.91	0.43	1.10 ±0.78
	1.47	1.37	1.30	1.38 ± 0.08
	1.88	1.13	0.78	1.26 ± 0.56
	1.52	1.09	0.96	1.19 ± 0.29
60-1	1.97	1.63	1.45	1.68 ± 0.26
	1.22	0.67	0.65	0.84 ± 0.32
	2.99	2.62	1.20	2.27 ± 0.94
	2.44	1.23	1.00	1.56 ± 1.77
	2.45	2.41	1.31	2.06 ± 0.64
60-2	1.93	1.83	1.18	1.64 ± 0.40

	1.72	1.65	1.08	1.48 ± 0.35
	3.13	2.52	0.84	2.16 ± 1.18
	2.29	1.88	1.85	2.01 ± 0.24
	2.65	1.11	1.71	1.82 ± 0.77

S4. Images of the capillaries with crystals.

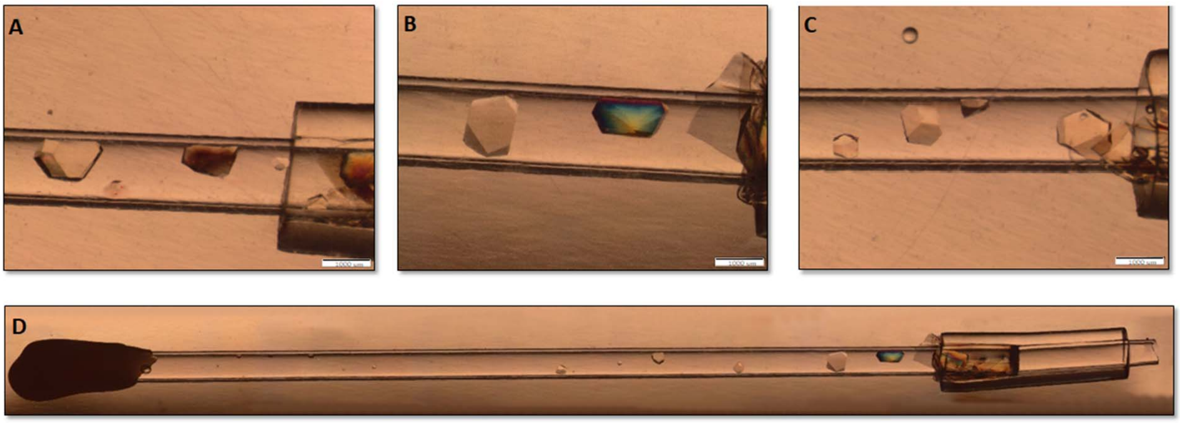

Figure S4.1 Representative image of crystalized XI, with initial protein concentration of 40 mg/ml, incubated at 4°C, (A) without a gel plug (B) with a 1 cm gel plug (C) with a 2 cm gel plug and (D) a representative image of the entire capillary with a 1 cm gel plug. The white scale in A to C is 1mm.
